# Ammonium chloride‐induced acidosis exacerbates cystitis and pyelonephritis caused by uropathogenic *E. coli*

**DOI:** 10.14814/phy2.15471

**Published:** 2022-09-23

**Authors:** Jeffrey M. Purkerson, Coralee A. Everett, George J. Schwartz

**Affiliations:** ^1^ Pediatric Nephrology University of Rochester Medical Center Rochester New York USA; ^2^ Strong Children's Research Center University of Rochester Medical Center Rochester New York USA

**Keywords:** acid–base physiology, inflammation, kidney collecting duct, urinary tract infection, uropathogenic *E. coli*

## Abstract

Acute pyelonephritis caused by uropathogenic *E. coli* (UPEC) can cause renal scarring and lead to development of chronic kidney disease. Prevention of kidney injury requires an understanding of host factors and/or UPEC adaptive responses that are permissive for UPEC colonization of the urinary tract. Although some studies have suggested urine acidification limits UPEC growth in culture, other studies have described acid‐resistance mechanisms (AR) in *E. coli* such as the CadC/CadBA module that promotes adaptation to acid and nitrosative stress. Herein we confirm and extend our previous study by demonstrating that despite urine acidification, metabolic acidosis induced by dietary ammonium chloride (NH_4_Cl‐A) exacerbates cystitis and pyelonephritis in innate immune competent (C3H‐HeN) mice characterized by: (1) markedly elevated UPEC burden and increased chemokine/cytokine and NOS2 mRNA expression, (2) accumulation of intravesicular debris noninvasively detected by Power Doppler Ultrasound (PDUS), and (3) collecting duct (CD) dysfunction that manifests as a urine concentration defect. Bladder debris and CD dysfunction were due to the inflammatory response, as neither was observed in Tlr4‐deficient (C3H‐HeJ) mice. The effect of NH_4_Cl‐A was unrelated to acidosis as dietary administration of hydrochloric acid (HCl‐A) yielded a comparable acid–base status yet did not increase UPEC burden. NH_4_Cl‐A increased polyamines and decreased nitric oxide (NO) metabolites in urine indicating that excess dietary ammonium shifts arginine metabolism toward polyamines at the expense of NO synthesis. Furthermore, despite increased expression of NOS2, NO production post UPEC infection was attenuated in NH_4_Cl‐A mice compared to controls. Thus, in addition to induction of metabolic acidosis and urine acidification, excess dietary ammonium alters the polyamine:NO balance and thereby compromises NOS2‐mediated innate immune defense.

## Graphical abstract

Acute pyelonephritis caused by uropathogenic *E. coli* (UPEC) can cause renal scarring and lead to development of chronic kidney disease. Excess dietary ammonium alters the polyamine:NO balance and thereby compromises NOS2‐mediated innate immune defense.

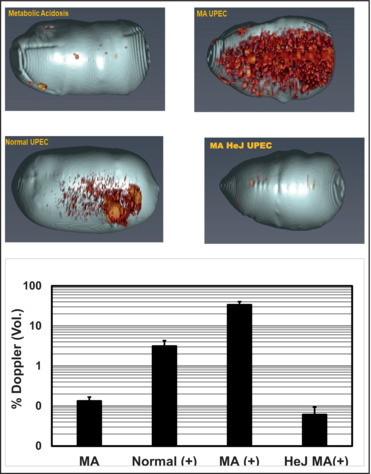

## 1 INTRODUCTION

Urinary Tract Infections (UTIs) are experienced by 1 in 3 women by the time they are 24 years of age (Foxman, 2002), and are particularly severe when they ascend from the bladder (cystitis) into the kidneys (pyelonephritis). In children the prevalence of acute pyelonephritis is due, at least in part, to vesicoureteral reflux (VUR) or retrograde flow of urine from the bladder to the kidneys (Fillion et al., 2014). Pyelonephritis‐associated societal expenses exceed 2 billion dollars annually (Brown et al., 2005). Pyelonephritis caused by infection with antibiotic‐resistant bacteria, such as uropathogenic *E. coli* (UPEC) has increased dramatically (Sun et al., 2020; Terlizzi et al., 2017) leading to concerns that the emergence of multi‐drug‐resistant microbial pathogens may ultimately render antibiotic therapies ineffective (Opal, 2016; Prabhu et al., 2013). Thus, antibiotic therapy alone is increasingly problematic for treatment of recurrent pyelonephritis; adjunct and/or replacement therapies need to be developed (Nordmann et al., 2011).

Manipulation of the host environment and/or UPEC adaptive responses that facilitate colonization of the urinary tract represents a key adjunct therapy aimed at reducing reliance on antibiotics. It has been reported that acidification of urine limits UPEC logarithmic growth in vitro (Paragas et al., 2014). The influence of metabolic acidosis and urine acidification on progression of UPEC‐UTI is incompletely understood. Due to regulation of systemic acid–base homeostasis by the kidney, urine exhibits a wide range of pH (4.5–7.5) whereas the pH of the blood (e.g. pH 7.36 to 7.44) remains relatively stable (Bilobrov et al., 1990; Schwaderer & Schwartz, 2004). Successful colonization of the urinary tract requires UPEC growth in acidified urine (Paragas et al., 2014), and UPEC resistance to acidification of endosomal and lysosomal compartments by V‐ATPase activity upon phagocytosis (Saxena et al., 2021). Thus, acid stress presents a substantial challenge to UPEC colonization of the urinary tract. In response *E. coli* have evolved acid resistance (AR) mechanisms such as the CadC/CadBA module that is comprised of: (1) CadC, an acid‐inducible transcriptional activator of the *cadBA* operon (Neely & Olson, 1996; Watson et al., 1992), (2) CadA, a lysine decarboxylase that produce cadaverine, a polyamine, and (3) CadB lysine‐cadaverine antiporter (Soksawatmaekhin et al., 2004) . UPEC resilience in acidified urine and/or intracellular compartments is mediated, at least in part, by the decarboxylation reaction that consumes one proton thereby increasing the UPEC cytoplasmic pH, and inhibition of porin outer membrane permeability by cadaverine (Samartzidou et al., 2003). Cadaverine as well as host‐derived polyamines (e.g. spermine and spermidine) also promote UPEC resistance to nitrosative stress (Bower et al., 2009; Bower & Mulvey, 2006). Induction of NOS2 (inducible Nitric Oxide Synthase, iNOS) leading to increased production of nitric oxide (NO) and reactive nitric oxide species (RNOS; nitrosative stress) is a key component of the TLR4‐dependent innate immune response to gram‐negative bacteria (Bogdan, 2015; Schairer et al., 2012). Thus, UPEC possess a metabolic program that promotes survival in acidified urine via amino acid metabolism, polyamines synthesis and transport, and detoxification of nitric oxide (Beebout et al., 2019; Beebout et al., 2021; Mason et al., 2009; Soksawatmaekhin et al., 2004).

Ammonium chloride (NH_4_Cl) supplementation in water or food has been extensively used in animal models to promote urine acidification and as an experimental model for studying the renal response to metabolic acidosis (McKinney & Burg, 1977; Nowik et al., 2010). Dietary ammonium induces metabolic acidosis (MA) by consuming bicarbonate during conversion of ammonia (NH_3_) to urea nitrogen via the urea cycle (Matsumoto et al., 2019). Our laboratory has extensively utilized NH_4_Cl administration as a model to study adaptation of kidney‐intercalated cells to metabolic acidosis (Purkerson et al., 2010, 2014, 2015), the impact of acidosis on HIF‐1α‐dependent antimicrobial peptide expression (Peng et al., 2017, 2019), and innate immune functions of α‐intercalated cells (α‐IC) (Hains et al., 2014; Saxena et al., 2018, 2019, 2021). Subsequent studies have focused on the metabolic acidosis induced by an ammonium chloride (2%)‐supplemented diet (NH_4_Cl‐A) on progression of UPEC‐UTI, and we recently reported that despite α‐IC activation and urine acidification, NH_4_Cl‐A, markedly impairs UPEC clearance and exacerbated pyelonephritis in innate immune competent C3H strains prone to vesicoureteral reflux (Purkerson et al., 2020). In the current study we confirm and extend these findings by demonstrating that NH_4_Cl‐A, but not acid‐loading via HCl‐supplementation of the rodent diet, impairs clearance of UPEC‐UTI. Exacerbation of pyelonephritis leads to CD dysfunction that manifests as a urine concentration defect. Studies present herein also support the hypothesis that NH_4_Cl‐A facilitates colonization of the urinary tract by attenuating Nitric Oxide Synthase 2 (NOS2)‐mediated innate immune defense by shifting arginine metabolism toward polyamine synthesis.

## 2 METHODS

### 2.1 Mice

C3H‐HeNCrl mice (Charles River, Wilmington, MA) and HeJ (Jackson Laboratory, Bar Harbor, ME) were purchased (age 4–5 week) and used for experimentation at 5–7 week of age. Mice were maintained on standard rodent chow (LabDiet 5010). All protocols and procedures involving mice were approved by the University Committee on Animal Rights of the University of Rochester Medical Center (UCAR‐2016–023).

### 2.2 Metabolic acidosis and assessment of acid–base status

Metabolic acidosis was induced via supplementation of rodent diet with 2% ammonium chloride (NH_4_Cl‐acidosis; NH_4_Cl‐A) ad libitum for up to 7 days. Ammonium chloride supplemented Labdiet® 5002 was formulated and manufactured by Purina Mills and distributed by Scott Pharma Solutions. HCl‐acidosis (HCl‐A) was induced by supplementing Labdiet 5010 with 1 ml/g 0.4 N HCl as described (Lee et al., 2009). Blood was collected from mice by tapping the retro‐orbital sinus under light anesthesia with a heparinized capillary pipette from which blood pH and s[HCO_3_^−^] were measured utilizing iSTAT® G3+ Cartridges (Abbott Labs). The volume and pH of dark cycle urine, collected under mineral oil from groups of 4–5 mice housed overnight in a metabolic cage with free access to food (powdered) and water, was determined the following morning. Urine pH was first estimated with pH strips (Whatman™Type CS pH indicator strips, 10SG Urine Reagent Strips; Thermo Fischer Scientific), and then confirmed using a calomel pH electrode (Table 1).

**TABLE 1:** Acid–base parameters

Condition	s[HCO_3_^−^]	N s[HCO_3_^−^]	Urine pH	N Urine pH
Normal	22.8 ± 0.7	13	6.9 ± 0.13	14
NH_4_Cl‐A	18.7 ± 0.6*	14	5.8 ± 0.05*	14
HCl‐A	16.3 ± 1.1*	4	5.7 ± 0.03*	5

*Note*: N _s[HCO3‐]_ = # blood collections 1/ms. N_urine pH_ = # independent urine collections (5 mice/metabolic cage). Average ± *SE*; *p < 0.001 vs. normal.

### 2.3 Urine collection and analysis

Urine was clarified by centrifugation at 3500× *g* for 10 min and then aliquoted and stored at −80 C until analysis. Urines were diluted 1:2 or 1:3 in distilled water for measurement of urine osmolality (mmol/kg) utilizing a Wescor 5500 Vapor Pressure Osmometer (Logan, UT). NO metabolites (NO_2_^−^ and NO_3_^−^) and total polyamines (TPA) in urine serial dilutions (e.g. 1:50–1:500) were measured via the Greiss assay and a fluorometric assay, respectively, utilizing commercially available kits according to manufacturer's recommended protocols (Sigma‐Aldrich). Urine polyamine and NO metabolite concentrations determined from standard curves were normalized to urine osmolality. Signal‐to‐noise in the TPA assay was improved by treating urine dilutions with 10–20 U urease (Type III from Canavalia ensiformis) for 1 h at 37 C as described by Webb‐Robertson et al. (2014). Urea depletion by urease treatment was confirmed by colorimetric detection of urea nitrogen utilizing a commercially available assay kit (ThermoFischer, Scientific). Statistical significance between groups was determined by TTEST (significance at *p* < 0.05). When three comparisons were performed, statistical significance was established as *p* ≤ 0.02 using the Bonferroni correction of the 95% confidence interval.

### 2.4 Uropathogenic *E. coil*

(UPEC) strain CFT073, an acute pyelonephritis isolate of *Escherichia coli* (ATCC® 700928™, Manassas, VA), and a stable transfectant of CFT073 expressing green fluorescent protein (UPEC‐GFP; kindly provided by Dr. Mathew Mulvey, University of UTAH) were used in this study. Stabs of glycerol (50%) stocks of UPEC strains stored at ‐80C were streaked onto tryptone‐phosphate‐agar plates and used to seed static UPEC cultures in tryptone phosphate broth. UPEC static cultures were transferred to a secondary static culture and after overnight incubation at 37 C, UPEC were washed in HBSS and UPEC colony‐forming units (cfu) were determined by measuring OD600nm (1_OD_ = 5 × 10^8^ cfu).

### 2.5 UPEC‐UTI

Mice were deprived of water 1 h before and after intravesicular inoculation of 10^7–8^ cfu in 50 ul HBSS via the transurethral approach as described (Hung et al., 2009). UPEC burden (UPEC cfu/g tissue) was determined by culture of serial dilutions of tissue homogenates in HBSS containing 0.025% Triton‐X‐100 for which UPEC clearance is defined as <50 CFU/g tissue (Hains et al., 2014). As bacterial burden is not normally distributed, statistical significance between groups was calculated with the Mann–Whitney *U*‐test (significance *p* < 0.05).

### 2.6 Live animal imaging via preclinical power Doppler ultra sonography (PDUS)

Bladder cystitis was imaged in mice anesthetized with ~2% isoflurane in oxygen, while resting on a thermostatically controlled surface to maintain body temperature, and after hair removal with a depilatory cream utilizing a Vevo 3100 preclinical ultrasound imaging platform. Quantitation of intravesicular Doppler signal (% Bladder Volume) was performed utilizing Vevolab 3.2.5 software (Visualsonics, Inc.) and 3D renderings of intravesicular debris (Doppler signal) were produced utilizing Amira 3D software (ThermoFischer Scientific).

### 2.7 Neutrophil depletion

Purified, in vivo grade (reconstituted in PBS 7.0 containing no preservatives or stabilizers) rat IgG2a isotype control antibody or monoclonal anti‐Ly6G (100 μg/100 μl, 1A8, BioXcell, Lebanon, NH) was injected I.P. on days −1, 0, and +1 with respect to transurethral inoculation with UPEC‐GFP (CFT073). Ly6G is expressed on myeloid cells including neutrophils (Privratsky et al., 2018; Rose et al., 2012; Yu et al., 2016).

### 2.8 Bladder/kidney gene expression analysis

First strand cDNA synthesis utilizing 100–500 ng of total RNA was accomplished using Superscript™ III First Strand Synthesis System (ThermoFischer). Relative abundance of mRNA's encoding GAPDH, AQP2, NOS isoforms, UT‐A (SLC14A2) chemokines (CXCL1,2, 5, and 12), cytokines(TNFα, IL‐1β, IL‐6) was determined by qRT‐PCR utilizing TaqMan™ gene expression master mix (ThermoFischer), TaqMan® Real Time PCR assays (FAM‐MGB probes, ThermoFischer Scientific; Table 2) and an ABI 7500 instrument (Applied Biosystems). Comparisons between groups were performed utilizing the ΔΔCt method and GAPDH as a reference gene. Statistical significance of fold changes in chemokine/cytokine mRNA abundance samples isolated from normal versus acidotic kidneys was determined by TTEST (significance at *p* < 0.05).

**TABLE 2:** TaqMan® real time PCR assays (ThermoFischer)

IL‐1β	TNFα	IL‐6	CXCL1	CXCL2
Mm00434228_m1	Mm00443258_m1	Mm00446190_m1	Mm04207460_m1	Mm00436450_m1
**NOS1**	**NOS2**	**NOS3**	**AQP2**	**SLC14A2**
Mm01208059_m1	Mm00440502_m1	Mm00435217_m1	Mm0437575_m1	Mm01261839_m1
**CXCL5**	**ATP6V1B**	**CXCL12**	**ADGRF5**	**GAPDH**
Mm00436451_g1	Mm00460309_m1	Mm00445553.m1	Mm01269030_m1	Mm99999915.g1

## 3 RESULTS

### 3.1 NH_4_Cl‐acidosis increases UPEC burden and NOS2 expression in the urinary tract

**FIGURE 1:**
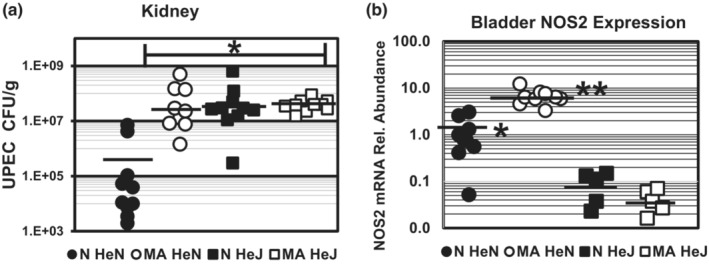
NH_4_Cl‐acidosis potentiates TLR4‐dependent induction of NOS2 mRNA expression during UPEC‐UTI. TLR4‐sufficient (C3H‐HeN) or TLR4‐deficient (C3H‐HeJ) were fed 2% NH_4_Cl‐diet (MA) or normal chow (N) Day −2 to Day +1–3 with respect to transurethral inoculation with UPEC (10^7–8^ cfu) on day 0. Panel (a) Kidney UPEC burden 3 dpi (Cfu/g tissue) was determined by serial plating of tissue homogenates. Bars denote median UPEC burden. *p < 0.01 vs. Normal HeN (N HeN) Mann–Whitney *U*‐TEST. Panel (b) Relative NOS2 mRNA abundance in bladder (1–2 dpi) was determined by qRT‐PCR via the ΔΔCt method utilizing GAPDH as a reference gene. *p < 0.01 vs. HeJ; **p < 0.01 vs. N HeN, TTEST. Bars denote average NOS2 mRNA relative abundance

Ammonium chloride acidosis (NH_4_Cl‐A) markedly impairs UPEC clearance and exacerbates pyelonephritis in innate immune competent (Tlr4‐sufficent) mice prone to vesicoureteral reflux (Purkerson et al., 2020). In experiments shown in Figure 1, C3H mice were administered either 2% NH_4_Cl‐diet or normal chow from day −2 up to 3 days post infection (dpi) with respect to transurethral inoculation of UPEC on day 0. Metabolic acidosis induced by the 2% NH_4_Cl‐diet characterized by reduced serum bicarbonate (18.7 ± 0.6 mM) and urine acidification (Urine pH 5.8 ± 0.05; Table 1). Kidney UPEC burden in TLR4‐sufficient C3H/HeN mice fed NH_4_Cl‐2%, was two to three orders of magnitude higher 3 days post infection (3 dpi) compared to mice fed normal chow (Figure 1a). Similar results were obtained for relative bacterial burden in bladder (not shown). Bacterial burden in the setting of NH_4_Cl‐A (HeN) was comparable to that of HeJ mice that do not effectively recruit neutrophils to the urinary tract during UPEC infection due to attenuated Tlr4‐signaling (Haraoka et al., 1999; Ragnarsdottir & Svanborg, 2012; Shahin et al., 1987). Administering the NH_4_Cl‐diet to HeJ mice did not further increase UPEC burden over Tlr4‐deficient HeJ mice fed normal chow (Figure 1a). Thus, NH4Cl‐A is not additive with TLR4 deficiency suggesting that NH_4_Cl‐A compromises some aspect of the TLR‐4‐dependent innate immune defense.

As induction of NOS2 (inducible Nitric Oxide Synthase, iNOS) leading to increased production of nitric oxide (NO) and reactive nitric oxide species (RNOS; nitrosative stress) is a key component of the TLR4‐dependent innate immune response to gram‐negative bacteria (Bogdan, 2015; Schairer et al., 2012), the effect of NH_4_Cl‐A on NOS2 expression in bladders of UPEC‐infected TLR4‐sufficient HeN versus TLR4‐deficient (HeJ) mice were compared. Relative abundance of NOS isoform (NOS1, NOS2, NOS3) mRNA in bladders harvested 1–2 dpi was determined by qRTPCR as described in methods. Consistent with an essential role for TLR4‐signaling induction of NOS2 expression during UPEC‐UTI, NOS2 mRNA abundance was >10 fold higher in normal HeN compared to Tlr4‐deficient HeJ mice (Figure 1b; mean ± *SE*: N HeN = 1.2 ± 0.34 vs. N HeJ 0.07 ± 0.03). NOS2 expression was significantly increased by NH_4_Cl‐A in normal infected HeN, but not TLR4‐deficient HeJ mice (mean ± *SE*: MA HeN = 6.7 ± 0.8 vs. MA HeJ 0.4 ± 0.01). Similar results were obtained for relative NOS2 expression in kidney, whereas NOS1 and NOS3 mRNA abundance was unchanged (not shown). Collectively, these results indicate that elevated UPEC burden in the setting of NH_4_Cl‐acidosis is associated with a selective increase in TLR4‐dependent NOS2 expression in the urinary tract.

### 3.2 Utilization of Power Doppler Ultrasound to detect cystitis caused by NH_4_Cl‐acidosis

**FIGURE 2:**
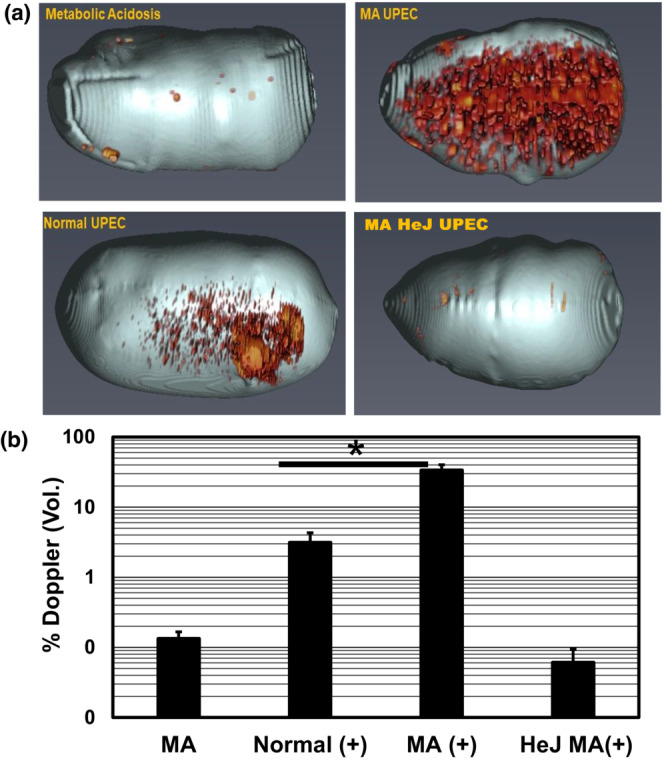
Non‐invasive detection of cystitis severity via PDUS imaging. Mouse bladders were imaged utilizing a Vevolab 3100 preclinical imaging system in Power Doppler Mode. Panel (a) Three dimensional renderings of intravesicular debris (Doppler signal) were produced utilizing Amira 3D software (ThermoFischer Scientific). Panel (b) The %Doppler signal with respect to bladder volume was measured utilizing Vevolab 3.2.5 software (Visualsonics, Inc.). MA = metabolic acidosis (NH_4_Cl‐A); HeJ = Tlr4‐deficient C3H‐HeJ mouse. *p < 0.02 vs. normal UPEC

**FIGURE 3:**
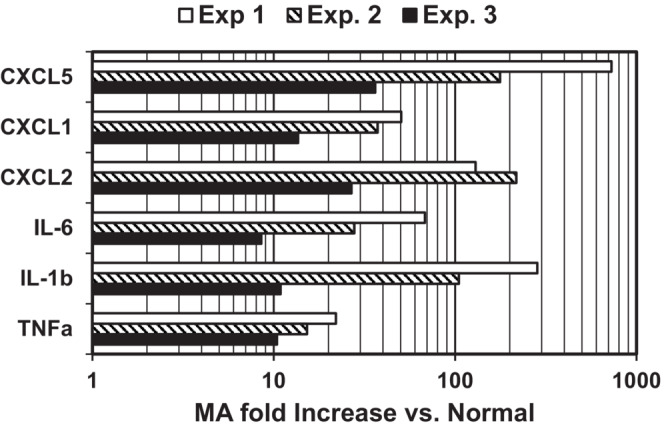
NH_4_Cl acidosis exacerbates bladder inflammation during UPEC‐UTI. Relative chemokine/cytokine mRNA abundance in bladder scrapes harvested 2–3 dpi were quantitated by qRT‐PCR with the ΔΔCt method utilizing GAPDH as a reference gene. MA = metabolic acidosis (NH_4_Cl‐A)

Quantitation of bacterial burden in bladder and kidney tissue is the standard approach for assessment of infection severity in mouse models of urinary tract infections (Hung et al., 2009). Tissue harvest is a terminal endpoint and thus large numbers of mice are required for studies aimed at monitoring progression and/or resolution of infection over time. Power Doppler Ultrasound (PDUS) is a non‐invasive imaging modality utilized to assess tissue and vascular structure–function relationships in health and disease (Martinoli et al., 1998). Concurrent with experiments shown in Figure 1, Power Doppler Ultrasound (PDUS) imaging was utilized to noninvasively assess cystitis severity 2–3 dpi in C3H mice prior to euthanasia for tissue harvest. Ultrasound imaging of infected mice experiencing NH_4_Cl acidosis in power doppler mode revealed motion of intravesicular debris (Figure 2a) that was quantitated as the intra‐vesicle % Doppler signal with respect to bladder volume measured utilizing Vevolab (Figure 2b) The Doppler signal was an order of magnitude higher in infected mice experiencing NH_4_Cl‐acidosis compared to normal infected controls (% Bladder volume: MA = 36.2 ± 7.8%; *N* = 4 versus Normal 3.1 ± 1.2%; *p* < 0.017, TTEST). The Doppler signal in uninfected normal or acidotic mice, and UPEC‐infected Tlr4‐deficient (HeJ) mice was negligible at <0.2% demonstrating that the Doppler signal was dependent on Tlr4‐ mediated inflammation associated with UPEC infection. The Doppler signal detects motion of intra‐vesicular debris that was likely composed of exfoliated urothelium and infiltrating neutrophils. The % Doppler signal was commensurate with the relative UPEC burden (Figure 1) and cytokine/chemokine mRNA expression in bladders isolated from UPEC infected control versus NH_4_Cl‐fed mice. In three independent experiments the relative chemokine/cytokine (IL‐1β, IL‐6, TNFα, CXCL1, CXCl2, CXCL5) mRNA abundance measured by qRTPCR in bladders isolated from mice fed excess dietary ammonium was increased from one‐two orders of magnitude (e.g. 8.5 to 727.6 fold) over normal infected controls (Figure 3). Collectively, these results demonstrated that PDUS can be utilized to non‐invasively assess cystitis severity in live mice.

### 3.3 NH_4_Cl‐acidosis‐induced pyelonephritis elicits a urine concentration defect

**FIGURE 4:**
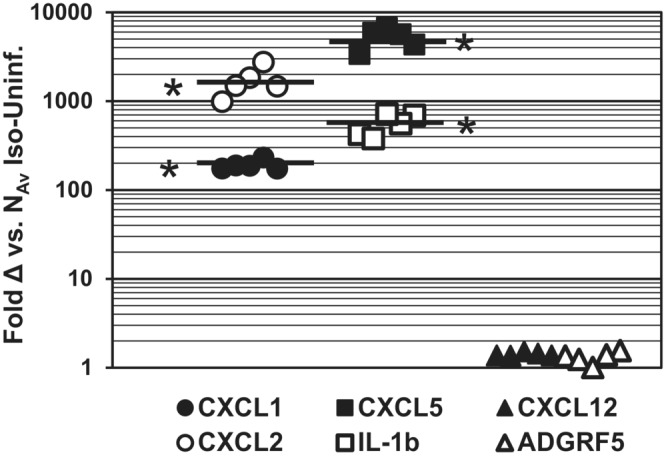
Transient Neutrophil Depletion exacerbates pyelonephritis. C3H‐HeN mice (5/group) were administered rat IgG2a isotype control (iso‐uninfect.) or monoclonal anti‐Ly6G (100 μg I.P.) days −1, 0,1 with respect to bladder instillation of 10^8^ cfu/50 ul UPEC strain CFT073 on Day 0. Kidneys were harvested 4 dpi and relative chemokine mRNA abundance in kidney wedges was determined by qRT PCR and the ΔΔCt method using GAPDH as a reference gene. Lines delineate average ΔΔCt with respect to N‐Iso, uninfected mice. *p < 0.001 vs. Iso‐uninfected, *t*‐test

Exacerbation of pyelonephritis by NH_4_Cl‐A also manifests as marked increase in chemokine/cytokine production within the renal CD (Purkerson et al., 2020). A urine concentration defect has been previously reported in rat models of experimental pyelonephritis (Gilbert et al., 1976; Kaye & Rocha, 1970), and thus whether NH_4_Cl‐A enhanced pyelonephritis triggered a urine concentration defect in the C3H model of UPEC‐UTI was examined. As shown in Table 3, urine output from UPEC‐infected HeN mice (2–4 dpi) experiencing NH_4_Cl‐A was more than double normal‐infected controls and was significantly increased over NH_4_Cl‐A alone. Furthermore, the osmolality of urine collected from UPEC‐infected NH_4_Cl‐A mice was nearly half the concentration observed in normal‐uninfected mice and was significantly reduced compared to mice experiencing NH_4_Cl‐A in the absence of infection. UPEC‐UTI induced increases in urine output and associated decreases in urine osmolality were not observed in Tlr4‐deficient HeJ mice (Table 3) indicating that the urine concentration defect was due to the Tlr‐4‐dependent inflammatory response. To determine whether perturbation of urine concentrating mechanisms by severe pyelonephritis was unique to the NH_4_Cl‐A model, the effect of exacerbation of acute pyelonephritis by transient neutrophil‐depletion was also examined. Partial neutrophil‐depletion was accomplished by administering monoclonal anti‐Ly6G (1A8, 100 μg I.P.) on Day −1, 0, and 1 with respect to transurethral inoculation of UPEC (10^8^ cfu) and dark cycle urine was collected 3–4 dpi prior to kidney harvest 4 dpi. We previously reported that partial myeloid‐cell depletion in this manner markedly elevates kidney UPEC burden (Purkerson et al., 2020). Transient neutrophil‐depletion in the setting of UPEC‐UTI caused severe pyelonephritis characterized by a marked increase in chemokine/cytokine expression (Figure 4). IL‐1β mRNA abundance measured by qRTPCR was 553 ± 67 fold increased in kidneys isolated from neutrophil‐depleted, UPEC‐infected mice relative to levels observed in kidneys from uninfected mice administered isotype control. Similarly, chemokines (e.g. CXCL1, CXCL2, and CXCL5) that play a pivotal role in neutrophil recruitment to sites of infection and injury (Rajarathnam et al., 2019) were induced 2–3 orders of magnitude, whereas CXCL12/SDF‐1 (Schwartz et al., 2015) and adhesion G‐protein coupled receptor F5 (ADGRF5, GPR116) expression was unaffected (Zaidman et al., 2020). Severe pyelonephritis in neutrophil‐depleted mice also resulted in a urine concentration defect that manifested as a doubling of urine output and significant decrease in urine osmolality compared to control mice (Table 3). Pyelonephritis in infected, neutrophil‐depleted C3H mice also resulted in an acid–base disturbance characterized by alkalosis concomitant with urine acidification (serum HCO_3_^−^ = 31.3 ± 0.6* mM, *N* = 6; Urine pH 6.55 ± 0.2; *p < 0.001 vs. Normal). Although investigating the mechanism(s) by which pyelonephritis attenuates urine‐concentrating mechanisms is beyond the scope of the current study, preliminary results suggest the urinary concentration defect is due at least in part to down‐regulation of AQP2 gene expression. During UPEC‐UTI AQP2 mRNA abundance determined by qRT‐PCR was significantly reduced in NH_4_Cl‐A and neutrophil‐depleted versus normal‐infected or uninfected isotype‐control mice, respectively (AQP2: Normal vs. Normal average (N vs. Navg) = 1.0 ± 0.2; NH_4_Cl‐A = 0.41 ± 0.09* and N vs. Navg =1.0 ± 0.1 Neutr. Depl. = 0.59 ± 0.05*; *p < 0.01 versus Normal; TTEST *N* = 5/group. In contrast, urea transporter (UT‐A; SLC14A2) and B1‐V‐ATPase (ATP6V1B) expression were not significantly changed by severe pyelonephritis UT‐A: NH_4_Cl‐A = 1.1 ± 0.5; *p* = 0.52; B1 (ATP6V1B): NH_4_Cl‐A = 0.85 ± 0.08; *p* = 0.12 versus N vs. Navg or 1.0 ± 0.2, *N* = 5/group). Collectively, these results demonstrate that NH_4_Cl‐A exacerbates UPEC‐UTI‐induced cystitis and pyelonephritis and that the latter results in kidney injury that manifests as a urinary‐concentrating defect.

**TABLE 3:** Pyelonephritis induces a urine concentration defect

Condition	Ur. Vol. (ml)/ms		Osm. mmol/kg	
	HeN	HeJ	HeN	HeJ
Normal	0.79 ± 0.05	0.57 ± 0.09	2730 ± 91	2612 ± 65
NH_4_Cl‐A	1.4 ± 0.11	1.03 ± 0.23	2300 ± 97	2449 ± 60
Normal‐UPEC	0.77 ± 0.14	0.25 ± 0.05	2489 ± 192	2849 ± 178
NH_4_Cl‐A UPEC	2.2 ± 0.47*	0.85 ± 0.85	1387 ± 163*	2907 ± 166
Neut. Depl‐UPEC	1.74 ± 0.06**		1440 ± 589**	

*Note*: Volume and osmolality of dark cycle urine collected via metabolic cage (5mice/group) were measured as described in Methods. Results are mean ± *SE* from 2 (Tlr4‐deficient HeJ) or 3–12 (Tlr4‐sufficient, HeN) independent urine collections.

* *p* < 0.01 vs. NH_4_Cl‐A.

** *p* < 0.03 vs. Normal‐UPEC.

### 3.4 HCl‐Acidosis does not affect UPEC burden in innate immune competent mice

**FIGURE 5:**
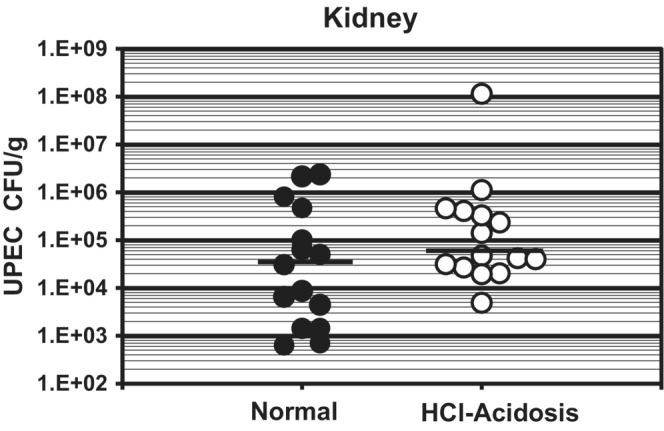
HCl‐Acidosis does not affect UPEC Burden in C3H HeN Mice. TLR4‐sufficient (C3H‐HeN) mice were fed HCl‐supplemented diet (1 ml/g 0.4 N HCl; HCl‐A) or normal chow (N) Day −2 to Day +3 with respect to transurethral inoculation with UPEC (10^7–8^ cfu) on day 0. Kidney UPEC burden 3 dpi (cfu/g tissue) was determined by serial plating of tissue homogenates. Bars denote median UPEC burden. *p* = 0.254 HCl‐A vs. normal, Mann–Whitney *U*‐test

Ammonium chloride (NH_4_Cl) supplementation in water or food has been extensively used in animal models to study the pathophysiology of metabolic acidosis (McKinney & Burg, 1977; Nowik et al., 2010); a 2% NH_4_Cl‐supplemented diet induces metabolic acidosis characterized by a significant reduction of serum bicarbonate concomitant with urine acidification (Table 1) However, despite activation of α‐IC mediated urine acidification (Purkerson et al., 2010, 2014, 2015), and upregulation of HIF‐1α‐dependent induction of antimicrobial peptide expression (Peng et al., 2017, 2019), results presented above indicate that NH_4_Cl‐A promotes progression of UPEC‐UTI. To determine whether UPEC colonization of the urinary tract is influenced by acid–base status independent of dietary ammonium supplementation, mice were administered rodent chow supplemented with 1 ml/g 0.4 N HCl to produce an acid–base state comparable to NH_4_Cl‐A (Table 1). HCl‐acidosis did not significantly change UPEC burden (Figure 5) suggesting that induction of acidosis per se does not explain the effect of excess dietary ammonium on UPEC colonization of the urinary tract, and urine acidification alone does not deter UPEC pathogenesis.

### 3.5 NH_4_Cl‐acidosis shifts polyamine: NO balance

**FIGURE 6:**
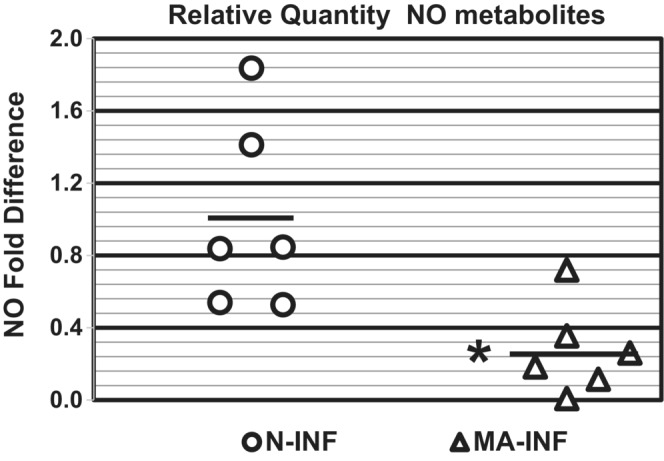
NH_4_Cl‐Acidosis attenuates NO production during UPEC‐UTI. Urine NO metabolites (NO_2_^−^and NO_3_^−^) were measured by the Griess assay were normalized to urine osmolality (5 mice/group). Normal chow (N) or NH_4_Cl‐A (MA). Fold difference vs. N_Avg_. Bars denote averages. *p < 0.05 vs. N‐Infected, *t*‐test

Ammonium generated via protein catabolism is converted to urea via the urea cycle, and blood urea nitrogen (BUN) is excreted by the kidneys (Matsumoto et al., 2019). L‐Arginine is the substrate for both arginase‐catalyzed synthesis of urea and NOS2‐catalyzed production of nitric oxide or NO (Caldwell et al., 2018; Munder, 2009; Rodriguez et al., 2017). As depletion of L‐arginine by arginase activity can lead to NOS uncoupling and decreased NO production (Caldwell et al., 2018; Rodriguez et al., 2017), the effect of NH_4_Cl‐A on NO production was determined by collecting dark cycle urine and measuring urine NO metabolites via the Griess assay (Giustarini et al., 2008). As shown in Table 4, NO metabolites normalized to urine osmolality were ~ 3‐fold higher in urine from HeN compared to HeJ mice fed normal chow suggesting constitutive regulation of NO production by Tlr4 signaling. In both HeN and HeJ mice the NH_4_Cl‐diet reduces urine NO metabolites by 37%–43% (Table 4), demonstrating that excess dietary ammonium attenuates NO production. Post UPEC infection NO metabolites in urine from NH_4_Cl‐A mice (HeN) were 26 ± 6% (i.e., 73.9 ± 6.4% reduction) of normal infected controls 1–3 dpi (Figure 6; Urine NO_3_^−^/NO_2_^−^: Normal diet 2.5 ± 0.5 mM; NH_4_Cl‐diet: 0.7 ± 0.3* mM; *N* = 6 collections 5 mice/collection *p < 0.02). In contrast, HCl‐acidosis did not significantly change urine NO metabolites as NO_3_^−^/NO_2_^−^ levels in urine from HCl‐loaded, UPEC‐infected mice were 86.2 ± 1.2% of normal (*n* = 4; *p* > 0.1). These results demonstrate that NH_4_Cl‐A markedly diminishes NO production during UPEC‐UTI despite upregulation of NOS2 expression (Figure 1b) indicating that NH_4_Cl‐A compromises NOS2‐mediated innate immune defense against UPEC‐UTI. Arginase catabolism of arginine yields ornithine, which in turn is metabolized to polyamines (e.g. putrescine, spermidine, and spermine; Caldwell et al., 2018). The effect of NH_4_Cl‐A on polyamine excretion was examined by measuring the concentration of total polyamines (TPA; normalized to urine osmolality) in urine collected via metabolic cage from mice fed 2% NH_4_Cl fed versus normal chow utilizing a fluorometric assay (see Section 2). As shown in Table 5, TPA were significantly increased by 54 ± 4.5% in mice fed the 2% NH_4_Cl‐diet for 2–3 days. In contrast, TPA in urine from HCl‐A mice were not significantly different from control mice (Data not shown). Thus, concomitant with reduced NO production (Table 4, Figure 6), NH_4_Cl‐acidosis increases polyamine excretion in urine and is associated with increased UPEC colonization of the urinary tract.

**TABLE 4:** NH_4_Cl acidosis attenuates NO production

Experimental parameters		Urine NO metabolites	
Strain	Condition	(NO_3_^−^, NO_2_^−^) mM	% Change
HeN	Normal	3.6 ± 0.58	−43.3%
	NH_4_Cl‐A	2.0 ± 1.1*	
HeJ	Normal	1.2 ± 0.02	−37.0%
	NH_4_Cl‐A	0.7 ± 0.04**	

*Note*: Mice were administered the 2% NH4Cl diet for 2–4 days and dark cycle urine was collected via metabolic cage (5 mice/group). Urine NO metabolites were measured by Griess Assay and normalized to urine osmolality. Results presented are Mean ± *SE* for 2–4 independent urine collections for each condition.

* p < 0.05 vs. Normal.

** p < 0.01 vs. Normal; TTEST.

**TABLE 5:** NH_4_Cl‐acidosis increases total polyamines (TPA) in urine

TPA (mM)	Normal	NH_4_Cl‐A	% Change
Exp. 1	2.4 ± 0.2	3.9 ± 0.9	63.1
Exp. 2	2.05 ± 0.4	3.04 ± 0.1	48.5
Exp. 3	1.7 ± 0.1	2.6 ± 0.3	51.3
Avg ± *SE*	2.1 ± 0.2	3.2 ± 0.4*	54 ± 4.5%

*Note*: Dark Cycle Urine was collected via metabolic cage (5 mice/group); Pre, Post = Before, after UPEC‐UTI; N = normal control; Total polyamines concentrations determined from a standard curve were normalized to urine osmolality.

* *p* < 0.05 vs. N_avg_.

## 4 DISCUSSION

In addition to induction of metabolic acidosis (Table 1), excess dietary ammonium attenuates a key facet of Tlr4‐dependent innate immune to UPEC‐UTI and thereby markedly exacerbates cystitis (Figures 2 and 3) and pyelonephritis (Figure 1) in C3H mice prone to VUR, (Purkerson et al., 2020), which in turn triggers CD dysfunction and a urine concentration defect (Table 3). Excessive dietary ammonium markedly enhances progression of UPEC‐UTI as UPEC burden in mice experiencing NH_4_Cl‐acidosis is 2–3 orders of magnitude higher than normal‐infected controls (Figure 1a) and UPEC burden in NH_4_Cl‐A mice was comparable to Tlr4‐deficient HeJ mice that do not mount an effective innate immune response to urinary tract infection by gram‐negative bacteria (Haraoka et al., 1999; Ragnarsdottir & Svanborg, 2012; Shahin et al., 1987). The effect of excess dietary ammonium is unrelated to acidosis, as induction of acidosis via HCl‐loading had no effect on UPEC burden in the C3H mouse model of experimental cystitis and pyelonephritis (Figure 5). NH_4_Cl‐A did not further increase UPEC burden in Tlr4‐deficient C3H‐HeJ mice (Figure 1a) in which NOS2 induction (Figure 1b) and neutrophil recruitment, (Haraoka et al., 1999; Ragnarsdottir & Svanborg, 2012; Shahin et al., 1987) are markedly attenuated, demonstrating the effect of excess dietary ammonium is not additive with Tlr4‐deficiency. Consistent with elevated UPEC burden (Figure 1a), NOS2 expression was markedly induced in infected mice experiencing ammonium chloride acidosis over normal‐infected controls (Figure 1b); however, NO production was markedly attenuated by excess dietary ammonium (Table 4, Figure 6). Collectively these results indicate that the excess dietary ammonium facilitates UPEC colonization of the urinary tract by attenuating NO2‐mediated innate immune defense.

A diagnostic tool that non‐invasively detects cystitis would facilitate the development of novel antibiotic‐sparing, adjunct therapies for urinary tract infections. Urine culture is the gold standard for microbiological diagnosis of UTI; however, diagnosis of UTI via urine culture is often inaccurate as this approach does not directly identify cystitis and thus can lead to misguided implementation of antibiotic therapy (McIsaac & Hunchak, 2011). Results shown in Figure 2 demonstrate that the magnitude of the bladder doppler signal detected by PDUS is commensurate with UPEC burden (Figure 1) and relative cytokine/chemokine mRNA expression (Figure 3) in the C3H model of UPEC‐UTI. The absence of the Doppler signal in Tlr4‐deficient HeJ mice during UPEC‐UTI demonstrates that Power‐Doppler UltraSound (PDUS) specifically detects bladder inflammation. The Doppler signal detects motion of intravesicular debris likely composed of exfoliated urothelium and infiltrating neutrophils in UPEC‐infected bladder. These results provide proof‐of‐concept for PDUS as a non‐invasive diagnostic tool for quantitative assessment of cystitis severity.

Exacerbation of pyelonephritis by NH_4_Cl‐A elicits a urine concentration defect characterized by a marked increase in urine output and a significant decrease in urine osmolality (Table 3). A pyelonephritis‐associated urine‐concentrating defect was not limited to the NH_4_Cl‐A models as severe pyelonephritis caused by transient neutrophil depletion in conjunction with UPEC‐UTI also triggered an increase in urine output concomitant with decreased urine osmolality. Renal inflammation rather than UPEC infection per se was causal, as the urine concentration defect was not observed in Tlr4‐deficient HeJ mice with high bacterial burden (Table 3; Figure 1a). In a mouse model of sepsis endotoxemia triggers a urine concentration defect caused by cytokine‐mediated down‐regulation of urea transporters that play an essential role in the urine concentration mechanisms within the distal nephron (Fenton et al., 2004; Sands, 2002; Schmidt et al., 2007). In contrast, urine concentration defect induced by UPEC‐UTI was associated with a nearly 3‐fold reduction in AQP2, but not UT‐A (SLC14A2), gene expression suggesting that pyelonephritis perturbs urine concentrating ability via a distinct mechanism(s). Results presented herein are consistent with the hypothesis that the urine concentrating defect caused by severe pyelonephritis is the result of excessive adenosine signaling in the CD. During unresolved inflammation adenosine can accumulate in tissues due to the release of adenine nucleotides from injured cells, which are converted to adenosine by the action of ecto‐nucleotidases (Linden, 2001). The urine concentration defect could be caused by adenosine signaling via A1 receptors that has been shown to reduce water reabsorption in the CD by blocking AVP‐mediated trafficking of AQP2 to the apical surface of principal cells and subsequent up‐regulation of AQP2 expression (Rieg & Vallon, 2009). Adenosine accumulation in renal medulla may also explain the pyelonephritis associated acid–base disturbance observed in C3H mice experiencing severe pyelonephritis due to attenuation of the neutrophil response (Figure 4). Signaling via adenosine receptors, ADORA2A or ADORA2B, stimulates proton secretion by α‐ICs (Battistone et al., 2018); thus adenosine production in pyelonephritis‐induced tissue injury may trigger dysregulated urine acidification leading to systemic alkalosis as is the case in GPR116 or ADGRF5‐deficient mice (Zaidman et al., 2020).

In contrast to NH_4_Cl‐A, acidosis induced via HCl‐loading had no effect on UPEC burden in innate immune competent C3H mice (Figure 5). As HCl supplementation of the rodent diet elicits an acid–base state comparable to NH_4_Cl‐A (Table 1), these results indicate that promotion of UPEC colonization of the urinary tract by NH_4_Cl‐A is independent of systemic acidosis. Furthermore, urine acidification in response to HCl‐loading did not facilitate UPEC‐UTI clearance. The latter result contradicts previous studies suggesting that UPEC colonization is limited by H^+^‐ATPase‐mediated luminal and intra‐vesicular acidification by α‐ICs (Paragas et al., 2014; Saxena et al., 2021). The ineffectiveness of HCl‐A in constraining UPEC‐UTI is likely due to the ability of UPEC to adapt to acid‐stress. *E. coli* encounter acid‐stress in virtually all colonized niche within the human host, and thus have evolved five acid resistance (AR1‐AR5) mechanisms (Bergholz & Whittam, 2007; Price et al., 2004). AR4 involves an *E. coli*‐derived polyamine, synthesized by an acid‐inducible lysine decarboxylase CadA. Cadaverine is a base and therefore buffers cytoplasmic pH in acidic environments. CadB lysine‐cadaverine antiporter exports cadaverine to the extracellular milieu (Soksawatmaekhin et al., 2004), where it inhibits porin mediated outer membrane permeability permitting UPEC survival in acidic niches (Samartzidou et al., 2003). Cadaverine synthesis in response to acid stress is regulated by CadC, an acid‐inducible transcriptional activator of the *cadBA* operon (Neely & Olson, 1996; Watson et al., 1992). Results presented herein are consistent with the hypothesis that activation of the *cadBA* operon by CadC enables UPEC colonization of the urinary tract during HCl‐acidosis (Figure 5), and likely enhances progression of UPEC‐UTI during NH_4_Cl‐A (Figure 1a).

Evidence presented herein demonstrates that NH_4_Cl‐A uniquely produces a host environment that is permissive to UPEC colonization of the urinary tract. An acid‐load (HCl‐A) and NH_4_Cl‐A produce a comparable acid–base state (Table 1); however, only metabolic stress induced by excess dietary ammonium selectively increases UPEC burden in Tlr4‐sufficient mice (Figure 1; Purkerson et al., 2020). NH_4_Cl‐acidosis attenuates NO production in C3H mice (Table 4), and despite elevated urinary tract NOS2 mRNA expression in the setting of NH_4_Cl‐A, NO production is markedly diminished compared to normal, infected mice (Figure 6). NH_4_Cl‐A also reduced NO production in HeJ mice (Table 4); however, NOS2 expression was not induced by UPEC‐UTI in HeJ mice (Figure 1b). Thus, the reduction of NO production by NH_4_Cl‐A is not additive with Tlr4‐deficiency and thus NH_4_Cl‐A does not further increase UPEC colonization of the urinary tract in HeJ mice. NOS2 and arginase compete for L‐arginine as a substrate and thus we hypothesize that by shunting L‐arginine metabolism via arginase and the urea cycle excess dietary ammonium reduces L‐arginine bioavailability for NOS2‐catalyzed production of NO (Caldwell et al., 2018; Munder, 2009).

In addition to weakening NOS2‐dependent innate immune defense, NH_4_Cl‐A increases excretion of host polyamines (Table 5), thereby shifting polyamine:NO balance in favor of polyamine synthesis. These results are consistent with the hypothesis that NH_4_Cl‐A attenuates NO production by shunting arginine metabolism via arginase as part of the urea cycle leading to increased polyamine synthesis (Caldwell et al., 2018; Munder, 2009). Elevation of urine polyamines is noteworthy as Mulvey and colleagues has shown that host (putrescine<spermidine≤spermine) and pathogen‐derived (e.g., cadaverine) polyamines enhance UPEC resistance to acid and nitrosative stress in vitro (Bower et al., 2009; Bower & Mulvey, 2006). Thus, the effect of NH_4_Cl‐A on progression of UPEC‐UTI highlights the pivotal contribution of host polyamine:NO balance to Tlr4‐dependent innate immune defense against UPEC colonization of the urinary tract and progression of UPEC‐UTI. Indeed preliminary studies suggest that inhibition of polyamine synthesis by administering Difluoromethylorthinine (DFMO), an ornithine decarboxylase inhibitor, facilitates clearance of a UPEC strain deficient in cadaverine synthesis (Bower & Mulvey, 2006).

Infection with antibiotic‐resistant bacteria has increased dramatically (Sun et al., 2020; Terlizzi et al., 2017) leading to concerns that the emergence of multi‐drug‐resistant microbial pathogens may ultimately render antibiotic therapies ineffective (Opal, 2016; Prabhu et al., 2013). Antibiotics are the most commonly prescribed drug for pediatric patients (Chai et al., 2012), and in addition to resistant bacteria, over‐exposure to antibiotics can lead to dysbiosis and predisposition toward an array of chronic diseases ranging from obesity to autoimmune disorders (Vangay et al., 2015; Wilkins et al., 2019). Studies presented herein support the hypothesis that pharmacological interventions targeting polyamine:NO in the host and/or adaptive responses of *E. coli* to acid and nitrosative stress may lead to the development of antibiotic‐sparing therapies for cystitis and pyelonephritis.
